# The Core of the Issue: Plank Performance and Pain in the Lower Back

**DOI:** 10.3390/jcm14113926

**Published:** 2025-06-03

**Authors:** Kira Eimiller, Leann LeFevre, Catherine Robarge, Cara Strano, Kelsey Tarbrake, Isabelle Wittmann

**Affiliations:** Department of Physical Therapy, Scott Bieler College of Health Professions, Daemen University, Amherst, NY 14031, USA; leann.lefevre@daemen.edu (L.L.); catherine.robarge@daemen.edu (C.R.); cara.strano@daemen.edu (C.S.); kelsey.tarbrake@daemen.edu (K.T.); isabelle.wittmann@daemen.edu (I.W.)

**Keywords:** low back pain, plank, supine bridge, anterior chain, posterior chain, core endurance, Oswestry, single-leg bridge, trunk stabilization

## Abstract

**Background and Objectives**: Low back pain (LBP) is a leading cause of disability worldwide. Core stabilization exercises such as the plank are often prescribed in rehabilitation settlings to improve neuromuscular control and spinal support. However, it remains unclear whether plank performance -accurately reflects trunk function or disability in individuals with LBP. The purpose of this study was to evaluate the relationship between plank endurance and low back pain in adults. **Methods**: A cross-sectional study was conducted with 117 adults aged 20–61 years (mean 26.0 ± 9.3), including both individuals with and without LBP. Participants completed a plank endurance test and the Modified Oswestry Disability Index (MODI). A subset of fifty-four participants with LBP also completed single-leg bridge tests to assess posterior chain endurance. Statistical analyses included Mann–Whitney U tests to compare plank times by LBP status, logistic regression to evaluate predictors of LBP, and correlation analyses to examine associations between the bridge-to-plank ratio and MODI scores. **Results**: Contrary to the initial hypothesis, individuals with LBP demonstrated significantly longer plank hold times than those without (U = 1861.00, *p* = 0.036). Logistic regression indicated that the overall model was statistically significant (χ^2^ = 12.39, *p* = 0.030), but plank duration was not an independent predictor of LBP (*p* = 0.070). Among participants with LBP, a higher bridge-to-plank ratio, reflecting relatively greater posterior chain endurance, was significantly associated with lower disability scores (Pearson r = −0.31, *p* = 0.023; Spearman ρ = −0.32, *p* = 0.018). **Conclusions**: These findings suggest that, while plank duration differs by LBP status, longer plank times may not indicate lower risk or severity of back pain. A greater balance of posterior chain to anterior core endurance may be more intricately linked to reduced disability, highlighting the importance of comprehensive core assessment and training strategies in rehabilitation.

## 1. Introduction

Low back pain (LBP) is currently the leading disabling condition worldwide, with incidence typically beginning in the third decade of life and rising steadily with age [1,2]. The most frequent source of mechanical LBP involves dysfunction of the lumbar spine, intervertebral discs, and associated soft tissues [3]. Contributing factors include altered motor control, muscular imbalance, degenerative changes, and psychosocial influences such as anxiety, depression, and job dissatisfaction, all of which complicate both diagnosis and treatment. The multifactorial nature of LBP presents a major clinical challenge, particularly given that recurrence rates one year after an initial episode range from 24% to 80% [1]. Globally, LBP contributes significantly to healthcare costs and work-related disability, representing one of the top causes of years lived with disability and lost productivity [1].

Impaired coordination of lumbar and core musculature—particularly asymmetrical activation and co-contraction—has been linked to mechanical imbalance and increased nociceptive signaling in individuals with LBP [2]. Effective segmental stability relies heavily on the coordinated recruitment of hip and trunk musculature, including both abdominal and back extensor muscle groups [4]. When muscular imbalances occur, especially between anterior and posterior chain muscles, lumbar stabilization is reduced, and symptoms of LBP may be exacerbated [4]. Beyond biomechanical factors, psychosocial contributors such as mood disorders and work dissatisfaction are also known to influence LBP risk and chronicity [1]. Resulting limitations in daily activity can impair social participation and overall quality of life [5,6]. Identifying specific functional impairments may support the development of more targeted and meaningful interventions.

Core stabilization exercises, such as the plank, are commonly prescribed for rehabilitation to improve lumbar motor control and segmental stability. In this study, core endurance refers to the ability of the trunk musculature to sustain isometric contractions over time, whereas core stability reflects the coordinated control of these muscles to maintain spinal and postural alignment during static or dynamic tasks. The Treatment-Based Classification (TBC) system recommends stabilization exercises for individuals with motor control or activation impairments [7]. While the 2015 revision of the TBC system emphasized the need for more clearly defined stabilization criteria, current clinical practice guidelines continue to support the use of targeted trunk muscle activation for both acute and chronic LBP [8]. 

Stabilization exercises aim to enhance spinal control through activation of core musculature [2,9]. However, individuals with LBP often exhibit delayed anticipatory activation of these muscles during postural tasks, contributing to compromised lumbopelvic stability and movement control [10]. These neuromuscular impairments raise questions about the clinical relevance of commonly prescribed exercises such as the plank and whether they sufficiently address the specific deficits in motor coordination observed in this population.

To examine whether core endurance reflects functional trunk control in individuals with LBP, plank duration was used as a measurable indicator. The prone plank involves lifting the body onto the forearms and toes, maintaining a straight alignment of the head, trunk, and lower limbs. The elbows are positioned under the shoulders, with scapulae retracted and hips aligned with the spine [11]. As this task requires sustained isometric activation of both deep and superficial core muscles, it may serve as a proxy for trunk stability and endurance in both symptomatic and asymptomatic individuals. Notably, surface EMG data suggest that the plank activates the rectus abdominis substantially more than the lumbar multifidus, raising concerns about its relevance for targeting deep spinal stabilizers [12]. Given these limitations, plank duration should be interpreted with caution as a measure of anterior core endurance, particularly in the absence of muscle activation or quality of movement data.

Given the high prevalence of LBP and the widespread use of core stabilization exercises in rehabilitation, it is important to evaluate whether commonly prescribed movements align with the functional deficits seen in this population. The plank is frequently emphasized in both clinical and fitness settings as a measure of core endurance and trunk control. However, its relevance as an indicator of functional core performance in individuals with LBP remains unclear. This study investigates the relationship between plank hold duration and self-reported disability in adults aged 20 years and older, with the goal of determining whether plank performance accurately reflects core function in the context of LBP. This study challenges the assumption that longer plank durations reflect better trunk function in individuals with LBP. It further explores the relationship between anterior and posterior trunk endurance, introducing the bridge-to-plank ratio as an exploratory metric to examine muscular balance.

**Hypothesis** **1.** *Individuals with LBP have significantly shorter plank times than those without LBP.*

## 2. Materials and Methods

This cross-sectional study included 117 adults (mean age 26.01 ± 9.34 years, range 20–61) recruited through convenience sampling via university email announcements. Participants completed a plank endurance test and were assessed for low back pain (LBP) using two measures: the Modified Oswestry Disability Index (MODI) and a binary self-report of LBP presence. The sample comprised 76 females and 41 males. Inclusion criteria were age ≥20 years and willingness to participate. Exclusion criteria included: (1) surgery involving the lumbar spine, hips, or knees within the previous six months; (2) physical trauma to the lumbar region or lower extremities within six months; or (3) current treatment for LBP by a healthcare provider.

Two separate data collection sessions were conducted: one including participants with and without LBP and a second including only participants with LBP. Participants, primarily university students and staff, completed a pre-participation questionnaire capturing weekly physical activity, height, weight, average sleep duration, prior injuries or surgeries, and LBP intensity on a 0–10 numeric scale. The sample included 76 females and 41 males. 

This study adhered to ethical standards for human research and was approved by the Institutional Review Board (IRB). Informed consent was obtained from all participants prior to enrollment. The primary investigator was a licensed physical therapist, board-certified in orthopedic physical therapy with 12 years of clinical experience. Four additional researchers were Doctor of Physical Therapy (DPT) students in the final year of their program and participated in data collection under faculty supervision.

**Figure 1:**
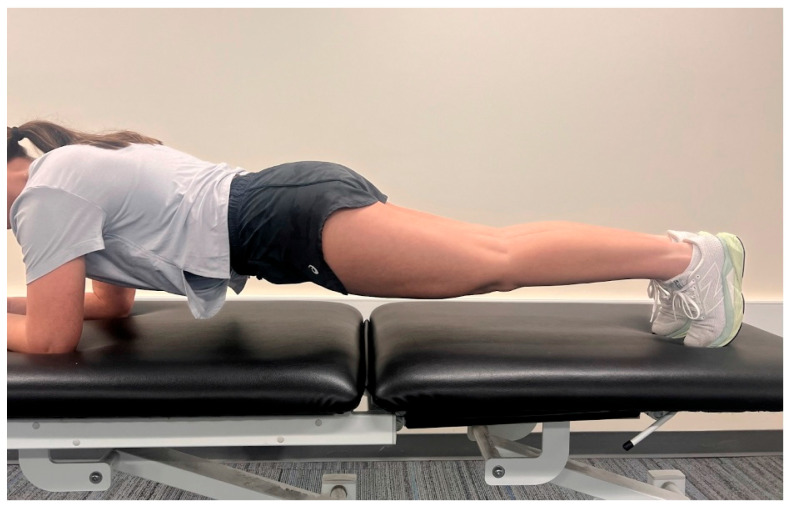
Prone Plank Test. Taken on iPhone13 Pro.

Following informed consent, participants completed the Prone Plank Test (aka prone bridge) as demonstrated in Figure 1 to assess core muscle endurance. Testing was performed on a firm exercise mat, with participants beginning in a prone position. Upon verbal instruction, participants elevated their body onto their forearms and toes, maintaining a straight alignment of the head, trunk, and lower limbs. Elbows were positioned directly under the shoulders, and participants were cued to maintain a neutral spine throughout the test. The test was terminated upon loss of neutral alignment (e.g., excessive lumbar extension or pelvic drop) or voluntary cessation. Plank duration was recorded in seconds using the built-in stopwatch application on an iPhone 13 (Apple Inc., Cupertino, CA, USA).

All endurance testing procedures were conducted by third-year DPT students who underwent standardized training in the administration of the plank and single-leg bridge protocols. Prior to data collection, raters practiced the procedures under faculty supervision to ensure procedural consistency. Importantly, the same student conducted all plank tests, and a second student conducted all single-leg bridge tests across participants, further minimizing inter-rater variability. While formal inter-rater reliability testing was not performed, all assessments followed a standardized script and timing method to promote consistency.

Only a single trial was administered for each participant to minimize fatigue and standardize testing. The test was conducted by a DPT student examiner who was separate from the screener and blinded to each participant’s LBP status. No practice trials were provided. Participants were instructed to perform a maximal sustained effort during the timed hold.

No formal warm-up was provided prior to the plank test. However, all participants completed four Modified Thomas Tests immediately beforehand as part of a concurrently conducted study. These tests were passive in nature and non fatiguing but may have introduced minor pre-test activity. All participants proceeded directly from these assessments to the plank test under standardized conditions.

**Figure 2:**
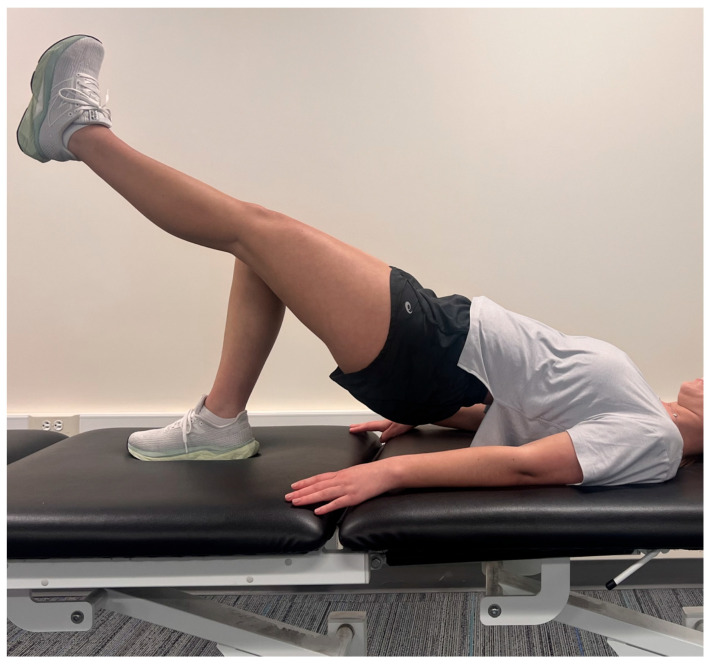
Single Leg Bridge Test. Taken on iPhone13 Pro.

Plank hold time, recorded in seconds, was treated as a continuous outcome variable. LBP status was evaluated using two methods: (1) a binary classification (presence or absence of LBP) based on participant self-report during screening, and (2) the Modified Oswestry Disability Index (MODI), a validated 10-item questionnaire used to quantify functional limitations related to low back pain [13]. Each item is scored on a 0–5 scale and converted to a percentage, with higher scores indicating greater disability. The MODI, a widely used adaptation of the original ODI, improves applicability by replacing the sexual activity item with one assessing employment or homemaking. It demonstrates strong responsiveness, with a minimal clinically important difference (MCID) of 6 points in individuals undergoing physical therapy [13]. A subset of 54 participants with LBP completed Phase 2 of the study, which involved performing a single-leg bridge test on both the left and right legs which is demonstrated in Figure 2. Participants were included in this analysis if they had valid data for single-leg bridge duration on both sides and a recorded plank hold time. A bridge-to-plank ratio was then calculated by dividing the average duration of the single-leg bridge holds (left and right combined) by the participant’s plank hold duration. This ratio was used to examine the balance between posterior chain and anterior core endurance.

### 2.1. Software and Assumptions

All statistical analyses were performed using SPSS (Version 28.0.0.0, IBM Corp, Armonk, NY, USA). Descriptive statistics were calculated for all variables, including means and standard deviations for continuous data and frequencies for categorical data. Normality of the primary outcome variables was assessed using visual inspection and the Shapiro–Wilk test. Because plank times were not normally distributed, nonparametric tests were used for group comparisons.

### 2.2. Group Comparisons

A Mann–Whitney U test was used to compare plank duration between participants with and without LBP.

### 2.3. Correlation Analysis

Spearman’s rank-order correlation was used to assess associations between variables that violated normality assumptions. Pearson correlation was included for supplemental comparison where appropriate. Among participants with LBP, the relationship between bridge-to-plank ratio and MODI score was specifically evaluated. Correlations were interpreted as small (r = 0.10–0.29), moderate (r = 0.30–0.49), or large (r ≥ 0.50). Statistical significance was set a priori at *p* < 0.05.

### 2.4. Logistic Regression

A binary logistic regression was conducted to examine whether plank duration predicted the presence of LBP. The model included five predictors based on theoretical relevance and preliminary group comparisons: plank duration (seconds), age (years), body mass index (BMI), weekly physical activity (hours), and weekly working hours. Odds ratios (ORs) with 95% confidence intervals were reported to quantify effect sizes.

### 2.5. Post Hoc Power Analysis

A formal a priori power analysis was not conducted. However, a post hoc sensitivity analysis using G*Power (version 3.1.9.7; Heinrich Heine University Düsseldorf, Düsseldorf, Germany) indicated that the total sample size (N = 117) provided approximately 75% power to detect a small to moderate group difference (Cohen’s d ≈ 0.37) in plank hold duration between individuals with and without low back pain at α = 0.05. This supports reasonable confidence in the observed between-group difference, while also indicating that future studies with larger samples may better detect smaller effects.

## 3. Results

A total of 117 participants (mean age 26.01 ± 9.34 years, range 20–61) were included in the analysis. The sample comprised 76 females (65.0%) and 41 males (35.0%). Most participants were university students (*n* = 97; 82.9%), while only a small proportion identified as athletes (*n* = 11; 9.4%). General demographic characteristics, including BMI, physical activity levels, sleep duration, and alcohol use, are summarized in Table 1.

**Figure 3:**
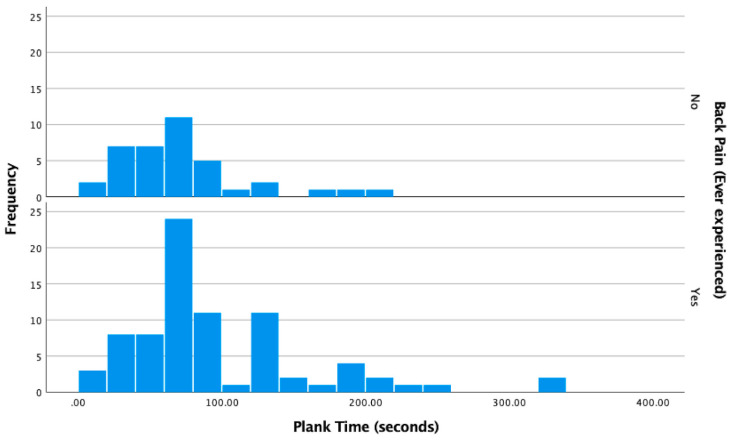
Distribution of Plank Duration by LBP Status.

A Mann–Whitney U test revealed a statistically significant difference in plank hold duration between participants with and without a history of low back pain (U = 1861.00, z = 2.096, *p* = 0.036). Contrary to the original hypothesis, individuals with LBP demonstrated longer plank durations than those without LBP. This finding suggests that increased anterior core endurance, as measured by the plank, may not be inherently protective against LBP. The distribution of plank durations by LBP status is illustrated in Figure 3. Frequency is expressed as the number of participants who held their plank for that duration of time. 

To aid interpretation, the rank-biserial correlation was converted to Cohen’s d, yielding a value of −0.435, which is a small to moderate effect size. Although the result was statistically significant, the modest effect size indicates that plank duration differences between groups account for only a part of the variability and that additional factors likely influence performance.

**Figure 4:**
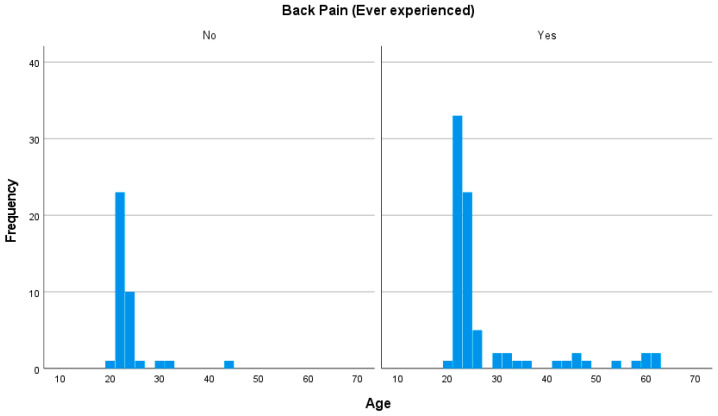
Histogram representing the frequency of age with correspondence to low back pain.

Age was examined as a potential factor associated with LBP status. A Mann–Whitney U test revealed a statistically significant difference in age between participants with and without LBP (U = 1910.50, *p* = 0.015), with individuals reporting LBP being significantly older on average. This finding is consistent with prior research linking age-related musculoskeletal changes and decreased physical activity with increased LBP risk. The distribution of age by LBP status is presented in Figure 4.

A chi-square test of independence was used to evaluate the association between gender and LBP status. No statistically significant relationship was seen (χ^2^ = 0.919, *p* > 0.05). While a slightly greater proportion of males (73.2%) than females (64.5%) reported a history of LBP, the difference was not statistically significant.

A binary logistic regression was conducted to examine whether plank duration, BMI, weekly physical activity, weekly working hours, and age predicted the presence of low back pain. The overall model was statistically significant (χ^2^ = 12.39, *p* = 0.030). None of the individual predictors reached statistical significance at the *p* < 0.05 level. However, plank time (B = 0.009, *p* = 0.070) and weekly working hours (B = 0.049, *p* = 0.071) showed small positive associations with LBP, with odds ratios of 1.009 and 1.050, respectively. Complete regression results are presented in Table 2.

**Figure 5:**
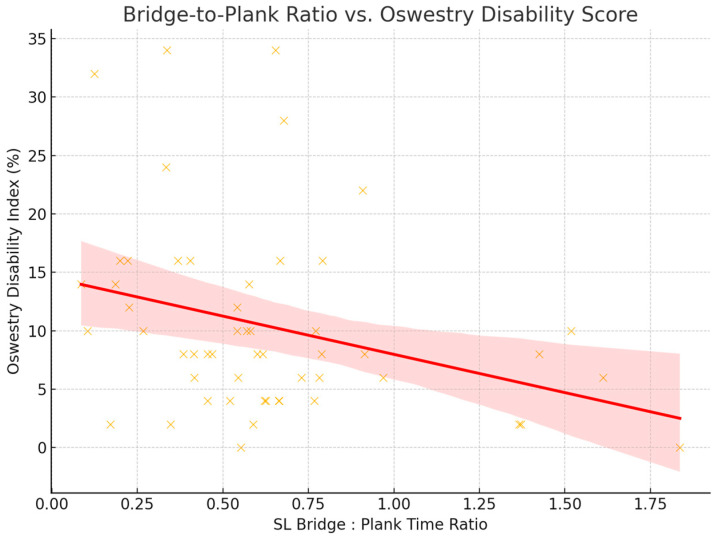
Scatterplot shows the relationship between Bridge-to-Plank Ratio and Modified Oswestry Disability Index (MODI) score among participants with LBP. Each point represents one participant. A significant negative correlation was observed (Spearman’s r = −0.31, *p* = 0.023), indicating that a higher ratio—reflecting relatively greater posterior chain endurance compared to anterior core endurance—was associated with lower reported disability. The red line represents the best-fit regression line with 95% confidence interval shaded in pink.

In a secondary analysis of 54 participants with LBP, a bridge-to-plank ratio was calculated to evaluate the balance between posterior chain and anterior core endurance. Pearson’s correlation revealed a significant negative association between the ratio and MODI scores (r = −0.31, *p* = 0.023), supported by Spearman’s rank correlation (ρ = −0.32, *p* = 0.018). The results indicate that participants with greater relative posterior chain endurance tended to report lower levels of perceived disability. The relationship is illustrated in Figure 5.

## 4. Discussion

This study examined whether plank hold duration differed between individuals with and without LBP. Contrary to the initial hypothesis, individuals with LBP held significantly longer plank times than those without, suggesting that increased anterior core endurance may not protect against LBP. Among participants with LBP, a higher ratio of posterior chain to anterior core endurance was associated with lower disability scores. Rather than undermining the importance of core strength, these findings align with prior work by McGill and others, emphasizing that neuromuscular coordination and muscular balance, particularly between anterior and posterior trunk musculature, may be more functionally relevant than isolated endurance in predicting outcomes related to LBP.

An unexpected finding was the inverse relationship between plank duration and LBP status. This challenges the assumption that longer plank times provide protection against spinal pain. Several hypotheses may account for this unexpected finding, although these remain speculative given the absence of neuromuscular data. Individuals with LBP may adopt compensatory strategies, such as increased use of superficial muscles or co-contraction, which can enable prolonged static holds but may reflect inefficient or maladaptive motor control rather than improved function [10,14]. These altered recruitment patterns may help stabilize the spine during isolated tasks like the plank while masking deficits in dynamic postural control and movement coordination. However, without EMG or movement analysis, such mechanisms cannot be confirmed. Future studies incorporating muscle activation data are necessary to clarify the underlying contributors to this observation.

Additionally, the plank primarily targets superficial anterior musculature, particularly the rectus abdominis, and provides limited engagement of deeper spinal stabilizers. Surface electromyographic (EMG) findings by Ekstrom et al. revealed that the rectus abdominis is activated 8.6 times more than the lumbar multifidus during the prone plank, highlighting a significant imbalance in muscle recruitment [12]. Without complementary assessments such as EMG or movement quality analysis, relying on plank duration alone may overstate anterior core endurance. This limitation reduces the plank’s utility as a standalone indicator of trunk stability and underscores the need for a multimodal approach to evaluating core function.

Furthermore, the plank is a static task that does not reflect the dynamic, multiplanar trunk control needed during functional activities [15,16]. As such, it may not adequately capture the neuromuscular coordination or motor control strategies that protect against LBP during movement [17]. This limitation is particularly relevant in young, physically active individuals, such as those in this study, who may have high performance on static tests despite underlying dysfunction. In these cases, the presence of LBP may not impair brief, maximal-effort tasks, masking deficits in dynamic control. Taken together, this suggests that plank duration alone may be insufficient as a proxy for core health or injury risk. Broader assessments that incorporate movement quality, motor control, and endurance balance across muscle groups may offer more meaningful insights into functional stability [17,18].

The results of this analysis suggest that the relative balance between posterior chain and anterior core endurance may be a crucial factor in perceived disability among individuals with LBP. Specifically, participants with higher single-leg bridge to plank time ratios, indicating relatively greater gluteal and hamstring endurance compared to anterior core endurance, tended to report lower scores on the Modified Oswestry Disability Index (MODI). This inverse relationship implies that individuals who have stronger posterior chain endurance relative to the anterior core may experience less functional limitation. Rather than viewing core strength in isolation, these findings highlight the importance of assessing muscular coordination and balance when evaluating trunk function when designing rehabilitation strategies for individuals with LBP. While the observed relationship between posterior chain dominance and lower disability was statistically significant, the effect size was modest. This aligns with the understanding that disability in individuals with LBP is multifactorial.

This evidence has meaningful implications for clinical rehabilitation. While planks are commonly prescribed to enhance trunk stability, the results suggest that a more balanced approach, emphasizing both anterior and posterior muscle groups, may be more relevant for individuals with LBP [9]. Specifically, a higher bridge-to-plank ratio was associated with lower disability, indicating that relatively greater posterior chain endurance may contribute to improved functional outcomes. This aligns with prior findings by Abdelraouf et al., who reported a strong negative correlation between trunk extensor endurance and back dysfunction in collegiate athletes with nonspecific LBP [19]. Their results reinforce the clinical relevance of targeting posterior chain musculature, particularly the trunk extensors, in managing LBP. This finding challenges the validity of using plank duration as a standalone indicator of core function. In some cases, individuals with LBP may maintain prolonged plank holds through compensatory bracing strategies or increased activation of superficial musculature, potentially masking underlying deficits in posterior chain engagement or dynamic trunk control [10,14]. These results support the integration of comprehensive core assessments that account for endurance balance and coordinated muscle activation when designing individualized rehabilitation programs for LBP.

Although age, weekly working hours, and BMI were not statistically significant predictors of LBP in the regression model, all three showed small positive associations. These trends suggest that cumulative physical stress, body composition, or age-related neuromuscular changes may contribute to LBP, even in younger populations. Their borderline influences call for further consideration when interpreting core performance and disability in diverse groups. The significant correlation seen between the bridge-to-plank ratio and lower disability scores aligns with prior research emphasizing the role of gluteal endurance and lumbopelvic stability in managing chronic LBP [4,20,21]. A higher bridge-to-plank ratio may reflect not only greater posterior chain endurance but also more efficient neuromuscular recruitment of the gluteus maximus and hamstrings, muscles critical for stabilizing the pelvis and limiting compensatory lumbar extension. This efficient posterior engagement may help reduce shear forces on the lumbar spine during movement and contribute to the lower self-reported disability seen in our participants.

It is important to emphasize that the results are correlational and derived from a subset of participants with LBP. While the observed relationships between endurance ratios and disability are clinically relevant, causality cannot be established. Moreover, this study did not assess individual variations in motor control strategies, muscle activation patterns, or movement quality, all of which may influence core performance and functional outcomes. Future research incorporating surface electromyography or motion analysis could offer deeper insights into the neuromuscular mechanisms underlying these endurance patterns and their association with perceived disability.

Nonetheless, the present findings reinforce a growing rehabilitation perspective that prioritizes integrated core training over isolated muscle strengthening. Emphasizing posterior chain development, particularly through exercises targeting the gluteus maximus, hamstrings, and lumbar extensors, may help reduce disability and enhance functional outcomes in individuals with LBP. Clinicians are encouraged to incorporate posterior chain endurance exercises, such as single-leg bridges, hip thrusts, or dynamic stability drills, alongside traditional anterior-focused movements to promote balanced trunk control and minimize compensatory movement patterns.

These findings support the use of comprehensive endurance assessments, such as the McGill Torso Endurance Test battery, which evaluates balance across anterior, posterior, and lateral trunk musculature. Compared to the plank alone, these tests may offer a more functionally relevant perspective on core stability and its relationship to LBP [19,22]. 

Knowing that chronic low back pain is associated with both delayed activation and structural changes in the trunk extensors, particularly the multifidi, rehabilitation strategies should aim to restore balance between anterior and posterior musculature. Fatty infiltration of the multifidus has been seen in individuals with chronic LBP, potentially reflecting atrophy or disuse and contributing to reduced motor control and stability [23,24]. Exercises that challenge postural control in positions with a decreased base of support, such as single leg bridging or bird-dog variations, promote co-contraction of anterior and posterior trunk musculature. Gatti et al. showed that such interventions can reduce pain and improve functional outcomes in individuals with chronic LBP [25]. This supports the use of targeted motor retraining that not only builds endurance but also restores coordination and recruitment patterns essential for dynamic trunk stability.

Chronic low back pain is often associated with fatty infiltration and delayed activation of the multifidi, which may impair anticipatory control and contribute to spinal instability and pain [23,24]. These neuromuscular deficits may limit the effectiveness of static endurance tasks and highlight the need for interventions that restore dynamic trunk control. Kumar et al. reported that dynamic exercises such as walking, stair climbing, and sit-to-stand tasks were significantly correlated with reduced pain and improved function in individuals with subacute or chronic LBP [26]. These findings support the integration of motor control retraining and functional movement-based interventions into rehabilitation programs to better address the underlying impairments contributing to LBP-related disability. This raises important questions about how different subgroups of LBP may respond to endurance-based assessments. Future research may benefit from stratifying participants by symptom duration (e.g., acute vs. chronic) or type (e.g., mechanical vs. radicular), as recommended in recent clinical guidelines [8], to better understand how specific subgroups differ in trunk muscle function and rehabilitation response.

While the bridge-to-plank ratio offers a novel perspective on muscular balance between the anterior and posterior chains, it has not been previously validated or standardized in the literature. No normative values or clinically meaningful cut-off scores exist to guide interpretation of this ratio. The bridge-to-plank ratio was not adjusted for gender or body size, which may have influenced individual performance. As such, our findings should be considered exploratory. Additionally, interaction effects between variables such as age and plank duration were not examined, which may have influenced the observed associations. Replication of this metric in larger, more diverse populations is warranted, along with investigation into its relationship with functional outcomes and neuromuscular coordination in individuals with low back pain. Larger and more diverse samples are needed to determine whether the bridge-to-plank ratio meaningfully reflects functional outcomes or neuromuscular coordination in individuals with LBP.

There are several limitations that should be considered when interpreting the findings. First, the cross-sectional design precludes causal conclusions on the relationship between core endurance, endurance ratios, and LBP. Second, the sample primarily consisted of young, physically active adults recruited from an academic setting, which may limit generalizability to older or more diverse populations. Future studies could aim to recruit from outpatient physical therapy clinics, community centers, or occupational settings to include older adults, individuals with chronic pain, and those with varying functional abilities. Such efforts would enhance the external validity and clinical relevance of core endurance findings in the context of low back pain. Third, LBP status and physical activity levels were self-reported and may be subject to recall bias. Additionally, plank and single-leg bridge times were used as proxies for anterior core and posterior chain endurance, respectively; however, these isolated tests may not fully capture the complexity of trunk motor control in functional tasks. Only a single plank trial was administered without familiarization. While this approach minimized fatigue, it may have introduced performance variability due to novelty, anxiety, or lack of warm-up. Also, pre-test factors such as recent physical activity, sleep, and nutrition were not controlled and may have influenced endurance performance as well as the potential of mild fatigue from the use of the Modified Thomas Test. Finally, psychosocial factors, known contributors to LBP, were not assessed and may have influenced both physical performance and self-reported disability outcomes. Constructs such as pain duration and fear-avoidance beliefs are strongly associated with the progression and functional limitation in individuals with LBP. The omission of validated measures such as the Fear-Avoidance Beliefs Questionnaire (FABQ) limits our ability to fully interpret participants’ performance and disability scores. Earlier research has shown that elevated fear-avoidance beliefs significantly predict greater disability and poorer work status outcomes in individuals with acute LBP [27]. Future research should incorporate such psychosocial assessments to provide a more comprehensive understanding of core function in individuals with LBP. Despite these limitations, the statistically significant results seen for plank duration differences, bridge-to-plank ratio correlations, and the overall logistic regression model suggest that the findings are unlikely to be attributable to random chance.

The present results contribute to an evolving research landscape that increasingly emphasizes multidimensional, mechanism-informed interventions for nonspecific low back pain. As highlighted in a recent bibliometric analysis, global research trends are shifting toward integrating exercise, disability metrics, and psychosocial factors such as fear-avoidance into rehabilitation approaches [28].

## 5. Conclusions

The findings of this study indicate that plank duration differs by LBP status; however, longer plank times were seen in individuals with LBP, challenging the assumption that greater anterior core endurance is protective. Contrary to the initial hypothesis, increased plank performance did not correspond with reduced disability. Although it is widely believed that individuals with LBP have reduced plank endurance due to impaired core muscle function, research findings do not consistently support this assumption. This assumption likely stems from documented delays in deep trunk muscle activation and coordination deficits among individuals with LBP [10,14]. However, several studies have failed to show significantly shorter plank durations in those with LBP [9,15,19], suggesting that static anterior endurance alone may not reflect meaningful functional impairment. At the same time, plank-based interventions have been shown to reduce pain and improve function [21,25], supporting their role in rehabilitation despite limited diagnostic value.

Instead, a higher bridge-to-plank ratio, reflecting relatively greater posterior chain endurance, was associated with lower disability scores. These results suggest that muscular balance and coordinated activation, rather than isolated anterior endurance, may be more relevant indicators of functional core stability. However, given the homogeneous nature of the sample of primarily young, physically active adults, the results should be interpreted with caution and may not generalize to older or more clinically diverse populations. Clinicians should consider comprehensive core assessments and training strategies that emphasize both anterior and posterior trunk musculature to better address the neuromuscular deficits associated with LBP.

## Figures and Tables

**Table 1:** General demographic table for the population being examined.

General Demographics									
		Age	Height (Inches)	Weight (lbs)	BMI (kg/m^2^)	Sleep (Hours per Night)	Working (Hours per Week)	Activity (Hours per Week)	Alcohol (Drinks per Week)
N	Valid	117	117	113	113	117	117	117	117
	Missing	0	0	4	4	0	0	0	0
Mean		26.01	66.91	162.35	25.41	6.75	11.07	5.94	2.60
Std. Deviation		9.34	4.19	32.18	3.75	0.87	13.82	4.70	3.63
Minimum		20.00	59.00	105.00	18.60	5.00	0.00	0.00	0.00
Maximum		61.00	78.00	258.00	36.10	10.00	60.00	30.00	24.00

**Table 2:** This table displays the relationship between participants who have LBP and their plank times (seconds), gender, activity (h/week), BMI (kg/m^2^), working hours (h/week), and age (years old).

Predictors of Low Back Pain								
							95% C.I. for EXP(B)	
	B	S.E.	Wald	Df	Sig.	Exp(B)	Lower	Upper
Plank time (seconds)	0.009	0.005	3.28	1	0.070	1.01	1.00	1.02
BMI (kg/m^2^)	−0.026	0.057	0.22	1	0.64	0.97	0.87	1.09
Activity (h/week)	0.048	0.066	0.52	1	0.47	1.05	0.92	1.19
Working (h/week)	0.049	0.027	3.26	1	0.071	1.05	1.00	1.11
Age	0.052	0.047	1.24	1	0.27	1.05	0.96	1.16
Constant	−1.35	1.76	0.59	1	0.44	0.26		

## Data Availability

Data are available from the corresponding author upon reasonable request.

## Abbreviations

The following abbreviations are used in this manuscript:

LBP	Low Back Pain
TBC	Treatment-Based Classification
EMG	Electromyography
ODI	Oswestry Disability Index
MODI	Modified Oswestry Disability Index
MCID	Minimal Clinically Important Difference
IRB	Institutional Review Board
DPT	Doctor of Physical Therapy
BMI	Body Mass Index
